# Hybrid Compression Optimization Based Rapid Detection Method for Non-Coal Conveying Foreign Objects

**DOI:** 10.3390/mi13122085

**Published:** 2022-11-26

**Authors:** Mengchao Zhang, Yanbo Yue, Kai Jiang, Meixuan Li, Yuan Zhang, Manshan Zhou

**Affiliations:** 1 College of Mechanical and Electronic Engineering, Shandong University of Science and Technology, Qingdao 266590, China; 2 College of Economics and Management, Shandong University of Science and Technology, Qingdao 266590, China; 3 College of Mathematics and System Sciences, Shandong University of Science and Technology, Qingdao 266590, China; 4 Libo Heavy Industries Science and Technology Co., Ltd., Taian 271000, China

**Keywords:** foreign object detection, belt conveyor, deep learning, computer vision, lightweight CNNs, network compression

## Abstract

The existence of conveyor foreign objects poses a serious threat to the service life of conveyor belts, which will cause abnormal damage or even tearing, so fast and effective detection of conveyor foreign objects is of great significance to ensure the safe and efficient operation of belt conveyors. Considering the need for the foreign object detection algorithm to operate in edge computing devices, this paper proposes a hybrid compression method that integrates network sparse, structured pruning, and knowledge distillation to compress the network parameters and calculations. Combined with a Yolov5 network for practice, three structured pruning strategies are specifically proposed, all of which are proven to have achieved a good compression effect. The experiment results show that under the pruning rate of 0.9, the proposed three pruning strategies can achieve more than 95% compression for network parameters, more than 90% compression for the computation, and more than 90% compression for the size of the network model, and the optimized network is able to accelerate inference on both Central Processing Unit (CPU) and Graphic Processing Unit (GPU) hardware platforms, with a maximum speedup of 70.3% on the GPU platform and 157.5% on the CPU platform, providing an excellent real-time performance but also causing a large accuracy loss. In contrast, the proposed method balances better real-time performance and detection accuracy (>88.2%) when the pruning rate is at 0.6~0.9. Further, to avoid the influence of motion blur, a method of introducing prior knowledge is proposed to improve the resistance of the network, thus strongly ensuring the detection effect. All the technical solutions proposed are of great significance in promoting the intelligent development of coal mine equipment, ensuring the safe and efficient operation of belt conveyors, and promoting sustainable development.

## 1. Introduction

In the modern coal mining industry, the belt conveyor assumes an important role as a “coal porter”. Benefitting from its advantages of low running resistance and continuous transport, belt conveyors have become the preferred equipment for coal transport in underground or opencast coal mines, and are currently developing in the direction of large-scale, intelligent, and energy-saving [1,2]. In detail, the intelligence of belt conveyors mainly means that the conveyor relies on various advanced sensors to realize its own self-perception of operation state, and make independent decisions to complete its own efficient operation with the optimal strategy and cooperate with the whole production process ideally, and it is also an important measure to guarantee the safe operation of belt conveyors [3].

However, due to the complexity of the working environment and the diversity of transport categories, the coal flow is often mixed with metal objects such as anchor rods, bolts, and iron sheets that are left behind, or large pieces of coal gangue that are mined from the upstream. These irregular objects can easily cause jamming of the chute, which will cause damage to the conveyor belt or even tear it directly, seriously threatening the safe operation of the belt conveyor and increasing the operation resistance of the belt conveyor, causing unnecessary energy waste [4,5]. Therefore, to reduce unnecessary damage to the conveyor belt and ensure its service life, it makes sense to identify and detect foreign objects embedded in the transported materials. This is a proactive measure to prevent conveyor belt damage, which nips potential risks in the bud [6]. Based on the above reasons and situation, many researchers have already carried out a lot of research work.

From the perspective of the detection method of conveying foreign objects, it can be divided into the manual detection and automatic detection methods. Among them, the manual detection method mainly relies on workers’ eyes to identify foreign objects, which is labor-intensive and only applicable to low-speed belt conveyors. Meanwhile, the automatic detection methods include the iron remover method [7,8], ray method [9,10], dielectric characteristic-based distinction method [11,12,13], machine vision method, etc. All these methods have their own limitations. In detail, the iron remover method is suitable for metal iron foreign objects, but is invalid for other foreign objects such as wooden sticks; the ray method has a certain degree of radiation, which is safe for human health. The dielectric characteristic-based distinction method is also limited to the distinction between coal and gangue, and cannot be used to distinguish other types of foreign objects. In contrast, the machine vision method benefits from its technical uniqueness of non-contact recognition and measurement, making it a good development in this field in recent years.

In detail, the detection methods based on machine vision can be divided into the traditional image processing-based method and deep learning-based method, according to the development process. Among them, the traditional image processing-based method is more commonly applied to the distinction between coal and coal gangue, which mainly uses the differences of targets in color, texture, shape, etc., as features, and then uses classifiers such as SVM to differentiate [14,15,16,17,18].

Compared with the traditional image processing-based method, the deep learning-based method is more commonly applied to the detection of more types of foreign objects. CK. Xiao et al. [19] have proposed a foreign object recognition method based on improved Faster Region-CNN (R-CNN) for coal gangue and iron flakes mixed in coal, and the detection accuracy has reached 95.35%; Wang Y et al. [20] further expanded the category of foreign objects to include four types: large coal, anchor, wood rod, and wood block. In addition, they used depthwise separable convolution instead of ordinary convolution in the backbone feature extraction network VGG16 of Single Shot MultiBox Detector (SSD) network, which reduced the number of parameters and improved the detection speed to a certain extent. Zhang K et al. [21] also attributed the categories of foreign objects to four types: rigid sticks, rigid planks, soft ropes, and soft cloth, and integrated depthwise separable convolution, attention mechanism, and classification activation map into Unet, which effectively improved the accuracy of foreign object detection on its self-made data set, as the detection accuracy has reached 97%. Further, from the algorithmic level, Ma G et al. [22] proposed to introduce depthwise separable convolution into a Centernet network to improve the detection speed of foreign object detection. The experimental data showed that the proposed algorithm can efficiently detect coal gangue, bolts, drill bits, and channel steel. Recently, Zhang MC et al. [6] prioritized the establishment of a conveying foreign objects dataset containing six types of foreign bodies, and further considered the higher real-time requirements of the target detection network for conveyors running at high belt speeds. They then introduced the Mobilenet series, Resnext series, and Shufflenetv2 series into the Yolov4 network, which effectively guarantees the detection accuracy and detection speed.

All the above schemes and methods have achieved good detection results in the laboratory, but there are still certain defects and deficiencies in practical application. Because the current video monitoring of belt conveyors in coal mines mostly uses network cameras for data collection, which do not have the ability to intelligently analyze, the collected data needs to be uploaded to the central server for centralized processing [23]. Due to the limitation of signal transmission bandwidth, there is a high network delay in this processing method, which has a great impact on the real-time processing and accuracy of the system early warning, and the simultaneous transmission and processing of multi-channel video data also requires a higher computing power of the central server [24,25].

In contrast with the centralized processing method, the edge computing method disperses the data processing tasks to the data acquisition end, thereby reducing the delay in the data transmission process and reducing the computing pressure of the central server, so it is more suitable for real-time. However, the computing power of edge computing devices is usually poor, and it is difficult for them to support complex deep neural networks. Therefore, to enable edge computing devices to have the same real-time processing capabilities, the corresponding algorithms or networks must be compressed and lightweighted to reduce the complexity and reduce the amount of computation. The existing methods of using compact convolution kernels instead of ordinary convolutions, such as using depthwise separable convolutions, have been proven to be time-consuming [26], so it is necessary to explore new acceleration methods.

In view of the current situation of conflict between the bottleneck of edge computing equipment or central server computing power and the high-performance network requirements of enterprises, this paper conducts research on an efficient identification and detection method of non-coal foreign objects based on hybrid compression optimization, aiming to use neural network compression techniques to lighten and improve the detection network for non-coal foreign objects, thereby compressing the number of parameters and the storage space occupied by the network model, and making the network less computationally intensive and faster in inference detection without losing detection accuracy, increasing its potential use in edge computing devices.

The remainder of the paper is organized as follows: Section 2 presents the relevant datasets and hardware configurations. Section 3 introduces the target detection network and its improvements, especially the principle and method of hybrid compression. The results of a case study and related discussion are presented in Section 4. Finally, the conclusions of the study are summarized in Section 5 and the future work is presented in Section 6.

## 2. Data Preparation and Hardware Basics

Data, computing power, and algorithms are the three essential elements of artificial intelligence. When both the computing power and algorithms (networks) have been determined, the quantity and quality of data directly determine the upper limit performance of deep learning target detection networks. Due to the particularity of the detection objects, there is no non-coal foreign objects dataset available for network training and testing in the existing public datasets. This study uses the same dataset constructed by researchers Mengchao Zhang and Yuan Zhang et al. [6]. In the dataset, there are 10,448 images with 31,288 labels, including 6 types of conveying foreign objects: coal gangue, wooden stick, bolt, iron sheet, angle iron, and iron rod.

The computing power is determined by the performance of the hardware. In this paper, three different devices that contain three CPU platforms (C1, C2, C3) based on different architectures and three GPU platforms (G1, G2, G3) are selected for algorithm or network testing, whose specific model can be clearly found in Table 1. Meanwhile, the algorithm-running environment and programming language usage are also given in detail in Table 1.

On the premise that the data and computational power are well determined, and to select a better target detection network for optimization, this paper gives priority to training a variety of target detection networks in the dataset, including the anchor-based one-stage target detection networks: SSD, Yolov3, Yolov4, Yolov5 [27], and also including the anchor-free-based target detection networks: CenterNet and YoloX. The performance of various networks in the dataset (on G2 platform) is shown in Table 2.

According to the results shown in Table 2, it can be found that the Yolov5-s network has achieved a higher prediction accuracy with the fastest prediction speed due to its fewer parameters, fewer calculation amount, and small model size occupation. Therefore, compared with other networks, the Yolov5-s network has a higher cost performance ratio, so we established Yolov5 as the main line to carry out the subsequent corresponding optimization work.

## 3. Network Structure and Hybrid Compression Optimization Method

### 3.1. Network Structure

**Figure 1:**
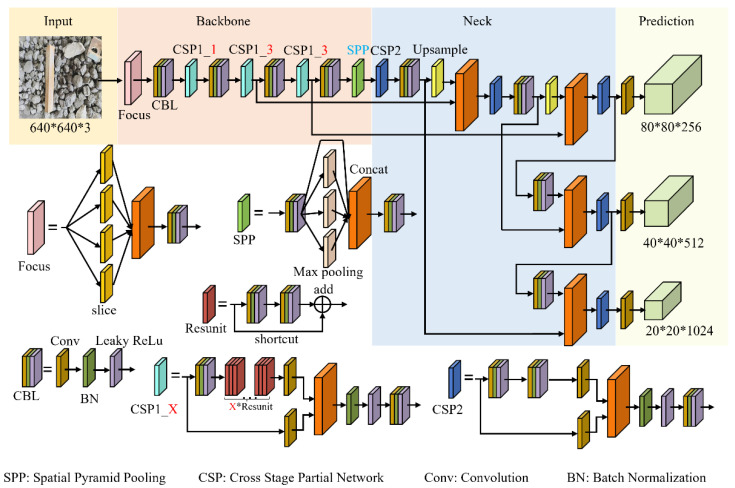
Structure of a Yolov5 network.

The structure of a typical target detection network usually consists of three parts: a feature extraction network, a feature enhancement network, and a prediction network. The former two are also called the backbone and neck, respectively. The prediction network uses the features extracted by the backbone and neck networks for prediction and regression to identify and locate the targets in the images. The structure of the Yolov5 network used in this paper is shown in Figure 1. Among them, the CSPDarknet network is used as the backbone, for which a large number of Cross Stage Partial (CSP) modules are used to increase the depth of the network to enhance the feature extraction capability of the network. Meanwhile, to avoid the gradient disappearance caused by the increase of network depth, in the CSP module many Resunit units are used to enhance the feature information, which further ensures the effective extraction of deep features. Furthermore, in the neck part of the feature enhancement network, upsampling and feature pyramid structure are used to fully integrate high-level semantic feature information with low-level location feature information, which makes up for the problem of less target location information in high-level feature information and effectively ensures the detection accuracy.

### 3.2. Hybrid Compression Optimization Method

**Figure 2:**
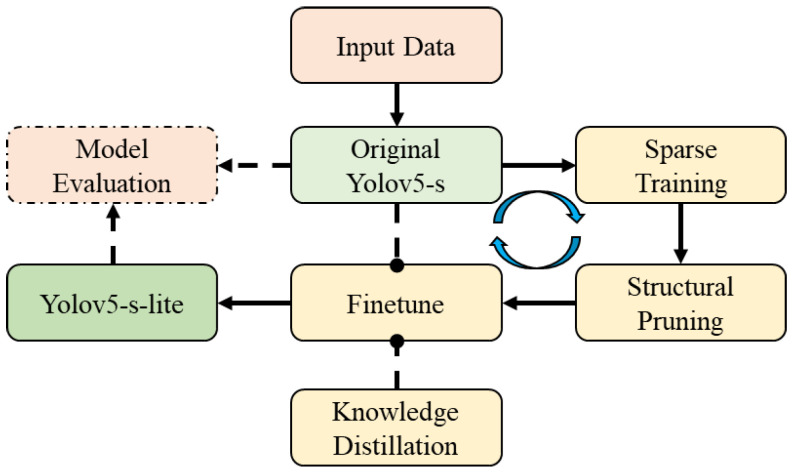
Process of the hybrid compression method.

Many studies have shown that the deep neural networks are facing severe over-parameterization, that is, there is a huge redundancy in the internal parameters of the network model, and deep neural networks may only need to train 5% of the parameters and use them to predict the rest of the network parameters, which can be comparable to the original ones [28]. Therefore, with the gradual transformation of deep neural networks from academia to industry in recent years, network compression and lightweight methods have gradually become a research hotspot. Among them, low-rank decomposition [29,30], sparse training [31], structure pruning [32,33,34,35,36], weight quantization [37,38], knowledge distillation [39,40], compact convolution kernel design, etc., have all proved to be very effective network compression methods. This paper proposes a hybrid compression optimization method that integrates sparse training, structural pruning, and knowledge distillation. The specific implementation process is shown in Figure 2, and it will be explained and described in detail below.

#### 3.2.1. Sparse Training and Structural Pruning

**Figure 3:**
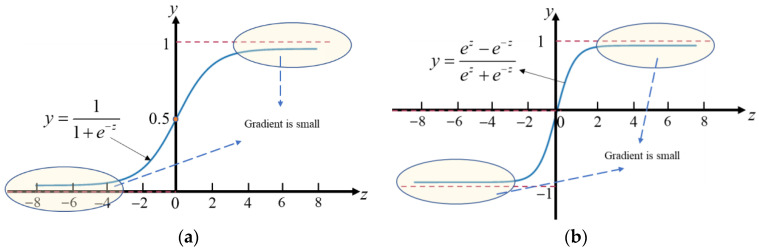
Gradient descent in activation function. (**a**) sigmoid activation function. (**b**) tanh activation function.

As the feature information is extracted by the deep neural network through the convolutional layer, its distribution will shift or change with the increase of network depth or training, generally towards the upper and lower limits of the value interval of the non-linear function. Taking the *sigmoid* and *tanh* activation functions as an example, as shown in Figure 3, the gradient of the neural network in the highlighted interval is very small, which is not conducive to the reverse neural network propagation, or will lead to slower convergence of the network, or will also lead to overfitting of the network.

Therefore, in the deep neural network, the combination of convolution layers, batch normalization (BN) layers, and activation function is generally used: the feature information extracted by convolution layers is preferentially adjusted by BN layers for data distribution, and then the nonlinear information will be introduced by the activation function, thereby improving the expressive ability of the neural network to the model; the specific structure can be found in the CBL module shown in Figure 1. In detail, the use of BN layers is to re-adjust the data distribution of the feature information of each channel extracted by the convolution layers to make it meet a relatively standard normal distribution, which ensures the result of each layer of the convolution calculation can be transmitted within an effective range, thus avoiding the disappearance of the gradient and speeding up the convergence speed. The specific implementation method is as follows:

Assuming that the input information of BN layers is a mini-batch consisting of *m* samples, $B=\left\{x^{\left(1\right)},\cdots ,x^{\left(m\right)}\right\}$, then the output result of batch normalization can be obtained by the following steps:

(1) Firstly, find the mean $\mu_{B}$ and variance $\delta_{B}^{2}$ of the mini-batch B:

(1) $$\mu_{B}=\frac{1}{m}\sum_{i=1}^{m}x^{\left(i\right)}$$

(2) $$\delta_{B}^{2}=\frac{1}{m}\sum_{i=1}^{m}{\left(x^{\left(i\right)}-\mu_{B}\right)}^{2}$$

(2) Secondly, standardize $x^{\left(i\right)}$ based on the above mean and variance:

(3) $${\hat{x}}^{\left(i\right)}=\frac{x^{\left(i\right)}-\mu_{B}}{\sqrt{\delta_{B}^{2}}}$$

(3) Thirdly, to ensure that the denominator in Equation (3) is greater than 0, a tiny constant ε greater than 0 is introduced, then Equation (3) can be corrected as Equation (4):

(4) $${\hat{x}}^{\left(i\right)}=\frac{x^{\left(i\right)}-\mu_{B}}{\sqrt{\delta_{B}^{2}+\varepsilon }}$$

(4) Further, since the standardized ${\hat{x}}^{\left(i\right)}$ will basically be limited to a normal distribution, which will reduce the expressive ability of the network, two learnable parameters could be introduced for further adjustment of the data distribution, namely the scaling parameter γ and the bias parameter β, both of which will be automatically updated when the neural network backpropagates for gradient descent. At this point, Equation (4) can be further corrected as follows:

(5) $${\hat{x}}^{\left(i\right)}=\gamma \times \frac{x^{\left(i\right)}-\mu_{B}}{\sqrt{\delta_{B}^{2}+\varepsilon }}+\beta$$

Through the above process, the BN layer could complete the batch normalization of the feature information of each channel, and then, through the activation function, more nonlinear information could be further introduced to the features. It can be seen from Equation (5) that when the scaling parameter γ is close to 0, no matter what the feature information of the channel is, the result of batch normalization will only be determined by the bias parameter β, and it has nothing to do with the channel feature information. Therefore, we can judge the importance of the network channel according to γ: in the training process, when γ approaches 0, the feature information of the corresponding channel will become useless information, and the network will become more sparse, which is the essence of network sparse training.

Since the scaling parameter γ and the bias parameter β are involved in the training process of the network, a penalty term can be added to the loss function to constrain it so that more of the γ converges to 0, thus achieving a greater degree of sparsity. In this paper, *L1* regularization is used as a penalty term added to the original loss function to drive the scaling parameter γ to approach 0 during training, resulting in a sparse network model. The new loss function after the penalty term added is shown in Equation (6).

(6) $$L_{n}=\sum_{\left(x,y\right)}l(f(x_{b},W),y_{b})+\lambda \sum \left|\gamma \right|$$

where $L_{n}$ denotes the loss function with the penalty term added; $\sum_{\left(x,y\right)}l(f(x_{b},W),y_{b})$ denotes the original loss function; $\left(x_{b},y_{b}\right)$ denotes the data and label provided by the dataset; W is the weights trained by the network; and λ is the regularization coefficient.

As shown in Equation (6), the loss function after adding the penalty term differs from the original loss function by taking |γ| into account. In detail, since the overall loss function $L_{n}$ is finally descended towards the direction of minimization, the value of |γ| is not allowed to increase; as the training progresses, the value of |γ| will gradually approach 0, which will lead to the failure or uselessness of the feature information of the corresponding channel and achieve the purpose of network sparseness. The value of the regularization coefficient λ determines the speed and constraint of network sparseness; the larger the value, the faster the network becomes sparse and the stronger the constraint.

Based on the sparse training of the network, the value of |γ| can be used as a criterion to measure the importance of the network channels. By pruning the channel where |γ| is closest to 0, a more lightweight network can be obtained. Further, since the calculation result of each channel in the output feature layer is also closely related to the filter, the judgment of the filter weight is also one of the factors to measure the importance of the channel. Therefore, we propose a channel importance scoring standard that comprehensively considers the BN layer scaling parameters and filter weights, as shown in Equations (7) and (8).

(7) $$E_{x}=\sum_{j=1}^{k}R(W_{j})$$

(8) $$m_{i}=\gamma_{i}\times E_{i}$$

where $E_{x}$ denotes the sum of the absolute values of the weights of filter *x*; k denotes the number of convolution kernels in the filter; $W_{j}$ denotes the *j*-th convolution kernel in filter *x*; $R(W_{j})$ indicates the *L1* norm of the convolution kernel $W_{j}$; $\gamma_{i}$ denotes the scaling parameter for the *i*-th channel; and $m_{i}$ is the importance score of the *i*-th filter.

The evaluation of the importance of each channel can be completed by Equation (8), and the comprehensive score set $M=\left\{m_{1},m_{2},\ldots ,m_{n}\right\}$ can be obtained. Finally, the pruning threshold of the filter in each convolutional layer can be obtained by Equation (9) and the preset pruning rate.

(9) $$\lambda_{pr}=sort_{pr}(M)$$

where $\lambda_{pr}$ is the pruning threshold; and $sort_{pr}(\cdot )$ denotes the ascending sort function, which will output the value at position *pr*.

Then, from the network structure of Yolov5-s shown in Figure 1, it can be found that the BN layers in the network are distributed in three positions, namely the CBL (Conv_BN_LeakyRelu) module used for channel number adjustment in the backbone, the CSP 1_X module used for network expansion in the backbone, and the CSP 2 module used for feature enhancement in the neck. This paper proposes three pruning strategies based on the distribution characteristics of the BN layer in the network, with the difference between them lying in the different processing ways of the BN layer in the CSP module.

(1). Pruning strategy 1:

In the structure of the CSP1_X module shown in Figure 1, there is a residual-like unit called Resunit, whose structure is also clearly given. From that, it can be clearly found that there is a residual edge (shortcut) and convolution layer splicing together through an add function for subsequent feature extraction. Due to the use of the add function, the two feature layers need to be consistent in dimension. Therefore, in pruning strategy 1, we do not perform pruning on the two convolution layers that are directly connected at the beginning and end of the shortcut to avoid dimensional processing.

(2). Pruning strategy 2:

Since pruning strategy 1 cannot sufficiently compress the network, in pruning strategy 2 we also prune the two convolutional layers that are directly connected to the shortcut. During the dimension processing, the number of remaining channels of the convolutional layer connected to the front end of the shortcut is taken as a reference to prune the channels of the convolutional layer inside the CSP module.

(3). Pruning strategy 3:

In pruning strategy 3, the pruning threshold will no longer refer to the importance score of the number of channels in each convolutional layer. Instead, after sparse training, a globally-based comprehensive score set is obtained based on Equation (8), and the pruning threshold is determined according to Equation (9) and the artificially set pruning rate. However, global pruning threshold cannot guarantee the integrity of some special network structures, such as residual blocks, so we also introduce a local security threshold to ensure the integrity of channel connections.

#### 3.2.2. Network Fine-Tune and Knowledge Distillation

As the pruning process breaks the structure of the original network, especially the number of channels in the convolutional layer, the pruned model cannot be directly used for prediction or performance evaluation. Therefore, it is necessary to fine-tune the remaining weight parameters to re-learn and iteratively update them in the dataset, so that they can re-learn the data feature distribution and achieve better prediction results.

The fine-tune process is the same as the normal training process, but the number of remaining parameters varies depending on the degree of pruning, so in some networks with a large degree of pruning, the effect of the fine-tune is not obvious because it is difficult to fit the data sample features well with a small number of parameters. In this paper, we propose to introduce the knowledge distillation strategy into the fine-tune stage and use the original unpruned Yolov5-s network as the teacher network to guide the pruned network to converge quickly and improve the prediction performance.

## 4. Results and Discussion

### 4.1. Results of Network Sparse Training

**Figure 4:**
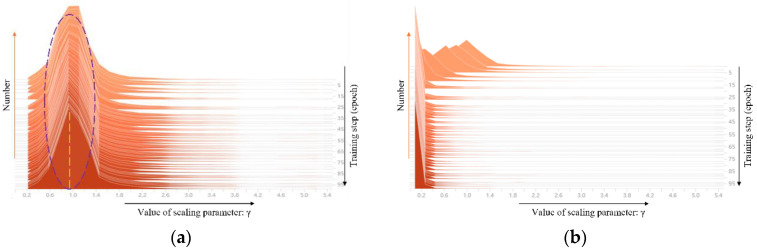

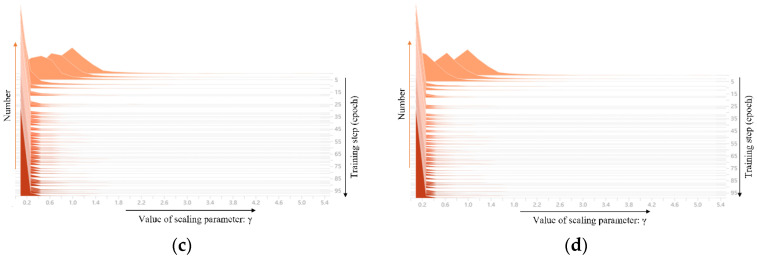
Distribution changes of scaling parameter γ with training under different sparsity factors. (**a**) *λ* = 0. (**b**) *λ* = 0.001. (**c**) *λ* = 0.0015. (**d**) *λ* = 0.002.

In this paper, the regularization coefficients λ are firstly set as 0, 0.001, 0.0015, and 0.002, respectively, and the original Yolov5-s network is sparsely trained for 100 epochs. Then, the distribution changes of scaling parameter γ during the training process are visualized in Figure 4. When λ=0, the distribution of scaling parameter γ basically presents a normal distribution with a mean value of 1.0, as shown in Figure 4a. In this case, there are very few γ at the 0 position, so it cannot be pruned. When λ=0.001, the distribution of scaling parameter γ gradually moves to the left from the top normal distribution and approaches 0 as the training progresses, which indicates that with the deepening of network training, L1 regularization as a penalty term has a good effect on the sparsity of the network and makes the difference of weight distribution of the BN layer more obvious, which provides a good criterion for channel and convolution kernel pruning. At the same time, from Figure 4b–d, it can be seen that as the value of λ increases, the training step size required to achieve the same degree of network sparsity decreases, which also confirms the correctness of Equation (6). Further, the results of sparse network training when λ=0.0015 are used for subsequent channel and convolution kernel pruning.

### 4.2. Results of Network Pruning

Three different pruning strategies are respectively adopted to prune the sparse Yolov5-s network, and the pruning rate ranges from 0.1 to 0.9. The effects of different pruning rates on network performance are respectively counted and shown in Table 3. Due to the damage of structural pruning to the network structure, it is difficult for the pruned network model and the corresponding weight to achieve high prediction accuracy. Therefore, in Table 3, we count more changes in the number of parameters and the size of the network model.

It can be seen from the above table that under the three pruning strategies, with the increase of the pruning rate, the parameters of the network scale and the size of the model show a significant downward trend: when pruning strategy 1 is adopted, as the pruning rate increases from 0.1 to 0.9 the amount of parameters decreases from 7.03 million (M) to 0.13 M, the parameter compression ratio reaches 98.15%, the corresponding network model size (weight occupied space) decreases from 13.7 MB to 0.35 MB, and the compression ratio reaches 97.44%. Similarly, when pruning strategy 2 is adopted, with the increase of the pruning ratio the parameter amount of the network model drops to 0.102 M, the compression ratio reaches 98.5%, the model size decreases from 13.7 MB to 0.28 MB, and the compression ratio reaches 97.9%. When pruning strategy 3 is adopted, the amount of network parameters drops from 7.03 M to 0.103 M, the compression ratio reaches 98.5%, the network model size drops from 13.7 MB to 0.28 MB, and the compression ratio reaches 97.9%. Then, by comparing the compression effects of the three pruning strategies it can be found that pruning strategies 2 and 3 have a more significant effect on the compression of parameters and model size.

**Figure 5:**
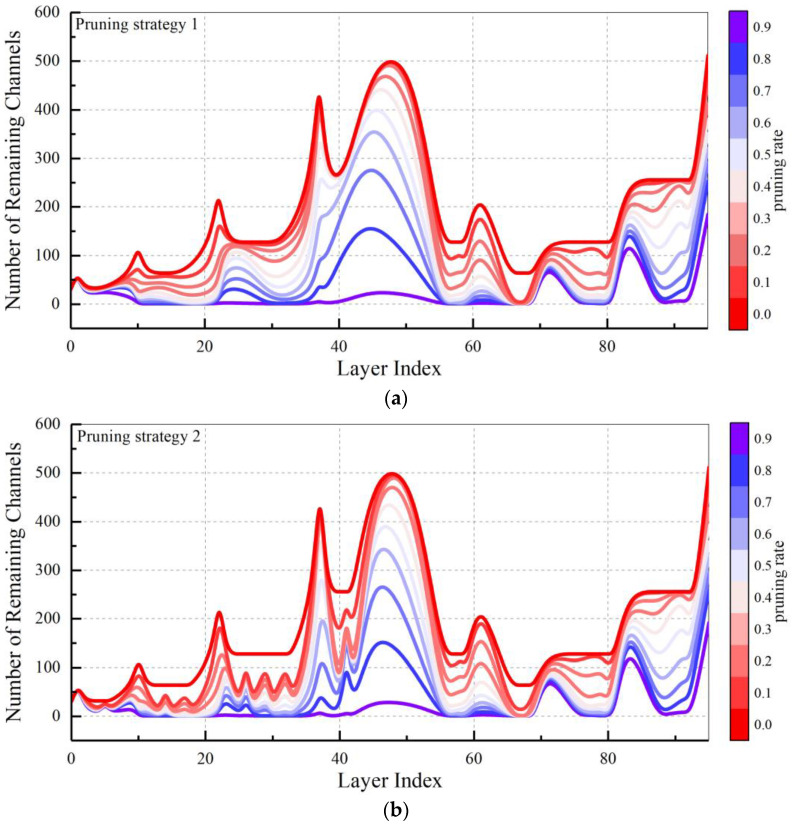

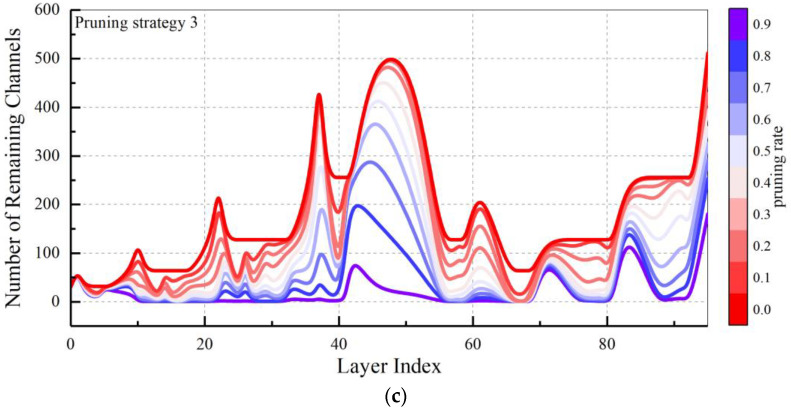
Effects of different pruning ratios and methods on the number of remaining channels (**a**) Result of pruning strategy 1. (**b**) Result of pruning strategy 2. (**c**) Result of pruning strategy 3.

Further, the number of remaining channels in each convolutional module under different pruning rate settings is counted separately in Figure 5. It can be found that under the three pruning strategies, the pruning of the convolutional channels is more concentrated in the front-end and middle layers of the network model, while the parameters of the deep few layers are retained after pruning. This is due to the prediction mechanism of the network. The deep network of the network needs to maintain a certain dimension for predicting the probability and location of the target. The distribution of the number of channels obtained after pruning shown in Figure 5 can also provide a reference for future simplified network structure design.

### 4.3. Results of Network Fine-Tune and Knowledge Distillation

In the fine-tuning stage of the network, the normal network fine-tuning method (FT 1) and the network fine-tuning method with knowledge distillation (FT 2) are used to retrain the pruned network for recovery and further testing of the network prediction performance. In FT 2, the original Yolov5-s network before sparse training is used as the teacher network, and the distillation temperature is set as 3 preferentially. Then, in the finetune process the total number of fine-tuning training steps (epochs) is set as 50, the batch size is set as 16, and two fine-tuning methods are counted, respectively. The effect of the two fine-tuning methods on the recovery of the network performance after pruning is counted separately, as shown in Table 4.

From the data shown in Table 4, it can be seen that at pruning rates of 0.1–0.5, both FT 1 and FT 2 have good recovery results for most of the pruned networks, and the effects of both are very similar; both can restore the performance of the pruned networks to close to that of the original networks, which fully proves that there is indeed some over-parameterization in the original networks, but also shows that under a small pruning rate, the effect of the fine-tuning with knowledge distillation method is not obvious. When the pruning rate is 0.6–0.8, the overall effect of the fine-tuning strategy becomes less and less obvious as the pruning rate increases, and when the pruning rate rises to 0.8 the network accuracy after fine-tuning still loses more than 5% compared to the original network; when the pruning rate further increases to 0.9, the difference between the effects of the two fine-tuning methods is particularly obvious: For pruning strategy 1, FT 1 can restore the pruned network accuracy to 58.4%, while FT 2, introducing the knowledge distillation strategy, can restore the network accuracy to 80.4%, which is 22% higher than that of the FT 1; For pruning strategy 2, FT 2 can improve the accuracy of the network by 3.6% compared with FT 1; For pruning strategy 3, the effect of FT 2 is relatively weak, with only a 2.9% improvement compared to FT 1.

**Figure 6:**
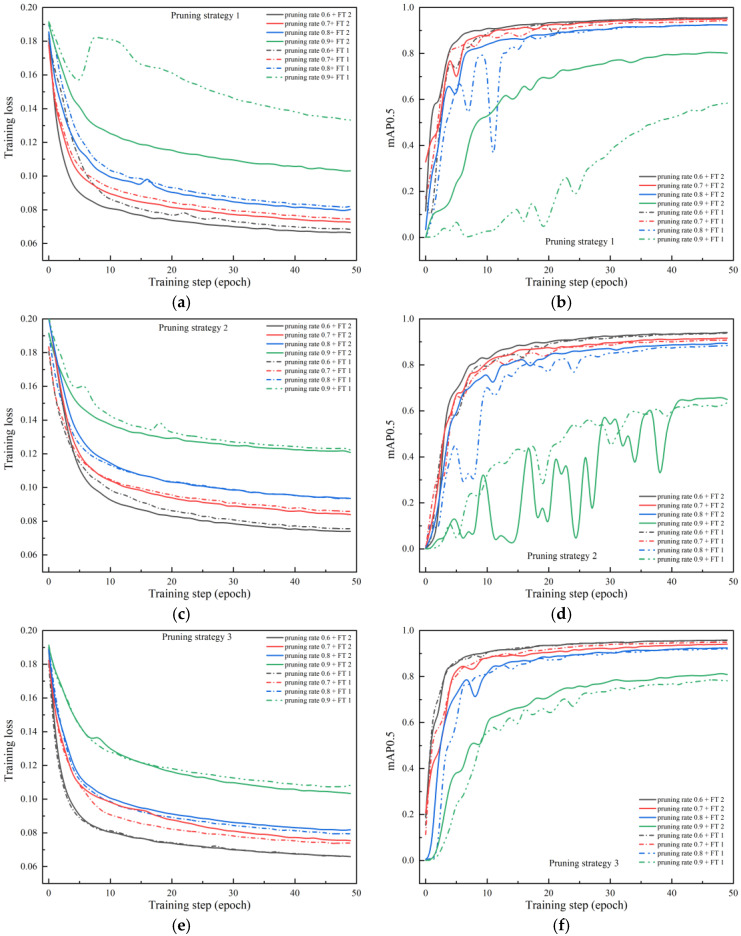
Loss and accuracy changes of three pruned networks under two fine-tuning methods. (**a**) Loss changes of pruning strategy 1. (**b**) Accuracy changes of pruning strategy 1. (**c**) Loss changes of pruning strategy 2. (**d**) Accuracy changes of pruning strategy 2. (**e**) Loss changes of pruning strategy 3. (**f**) Accuracy changes of pruning strategy 3.

The data in Table 4 can represent the result of network fine-tuning, but cannot accurately reflect the dynamic adjustment process of the re-training process. Therefore, we further visualize the training loss and accuracy changes of the network with different fine-tuning methods for pruning rates of 0.6 to 0.9, as shown in Figure 6.

By comparing the fine-tuning results of the network under different pruning rates in Figure 6, it can be found that: (1) when the pruning rate is 0.9, the training loss of the network in the fine-tuning process is significantly higher than that of other pruning rates, and the prediction accuracy mAP is also obviously lower than that of others; and (2) the network fine-tuning method with knowledge distillation can make the loss reduction process more stable, and can accelerate the convergence of the network to a certain extent, thereby reducing the training cost.

By observing the changing trend of different pruning strategies in the network fine-tuning process, it can be found that when the network obtained by pruning strategy 2 is fine-tuned, the training loss and accuracy of the network have great fluctuations, especially when the pruning rate is 0.9; while the training loss basically presents a steady declining trend, its accuracy on the validation set has huge fluctuations, which means that the current network may be in the following two states: either the network is in a state of overfitting where network generalization ability is poor, so the training set weight does not fit the validation set of data distribution; or the network is in a special form of “owe fitting” where only a small amount of parameters in the network are not enough to fit the characteristics of the data distribution, and as some of the channel numbers have fallen to a minimum of 1, each fall in vectors based on tiny iteration will also lead to sharp fluctuations in the performance of the network, and so cannot be fine-tuned to have a very good training effect. According to the actual scenario in this paper, the situation in this paper belongs to the second state, that is, the network is in the state of underfitting, and excessive pruning leads to the reduction of the network capacity and the deterioration of generalization ability, which is not suitable for use in the future.

### 4.4. Inference Speed Test of the Network

The pruned networks recovered some of their performance after fine-tuning, and could be further used to test the inference speed of the networks since the different fine-tuning methods affect the accuracy of the network but not the number of parameters or the inference speed. Then, the computation and inference speed of the improved network model with different pruning rates for the three pruning strategies are compared in Table 5 and Table 6, on the different hardware displayed in Table 1 (G1: GTX 1650-4G, Turing architecture; G2: RTX 2060-6G, Turing architecture; G3: RTX 3080-10G, Ampere architecture). All tests on inference speed are the averages obtained by repeating the experiment more than 900 times.

Table 5 shows the computation statistics of the network model after pruning; it can be found that as the pruning rate increases, the computation of the network shows an obvious decreasing trend. For pruning strategy 1, the network computation amount drops to 2.5 GFLOPs when the pruning rate is 0.9, which is a decrease of 84.9%. For pruning strategy 2, the computation is the lowest among the three pruning strategies, at 1.9 GFLOPs with a decrease of 87.5%. Then, for pruning strategy 3, the computation of the network model is 2.2 GFLOPs at a pruning rate of 0.9, a decrease of 85.5%. In summary, it shows that the pruning strategy proposed in this paper has a significant effect on the compression of the network model computation.

As for the detection speed, the test data of the fine-tuned networks on GPUs shown in Table 6 show that the pruned networks achieved a certain degree of improvement in inference speed on three different hardware bases. On G1, pruning strategy 3 achieved the fastest inference speed of 88.9 FPS at a pruning rate of 0.9, which is 70.3% faster than the inference speed of 52.2 FPS achieved by the original network without pruning. On G2, pruning strategy 3 achieved the fastest inference speed of 72.2 FPS at a pruning rate of 0.5, which was 52.2% and 68.7% higher than the original network’s inference speed of 65.3 FPS. On G3, thanks to the powerful hardware performance, firstly, the inference speed of the original network was increased to 148.1 FPS, and then pruning strategy 3 achieved the fastest inference speed of 176.2 FPS at a pruning rate of 0.9, which is a 28.1% improvement compared to the original network.

Comparing the performance of the three pruning strategies on three different hardware (GPU), it can be found that, compared with pruning strategies 1 and 2, pruning strategy 3 has a more significant effect on speeding up network inference. At the same time, it can also be proven that the same network model has different inference speeds on different hardware architectures and platforms, and the lightness of the network cannot be measured by the number of parameters and the amount of calculation alone. This is consistent with the conclusion obtained in ShuffleNet v2 [26]: the inference speed of the network was not only affected by factors such as the amount of computation and parameters, but also limited by the memory access cost (Mac) and the degree of parallelism of chip processing. Therefore, in Table 7 we continue to compare the inference of the networks obtained by the three pruning strategies on three different CPU hardware, also given clearly in Table 1 (C1: E5-2620V3, Haswell E architecture; C2: i7-9750H, Coffee Lake architecture; C3: i5-12400F, Alder Lake-H architecture).

From the results shown in Table 7, it can be found that with the increase in the pruning rate, the inference speed of the network obtained by the three pruning strategies on the three different CPUs is steadily improving, which basically shows a positive correlation with the pruning rate, and both achieved the fastest inference speed when the pruning rate is 0.9. On C1, pruning strategy 2 achieved the fastest inference speed of 10.3 FPS, compared with 4.0 FPS achieved by the original network, and the inference speed is improved by 157.5%. On C2, pruning strategy 2 achieved the fastest inference speed of 8.9 FPS, an increase of 128.2% compared to 3.9 FPS. On C3, it is still pruning strategy 2 that achieved the fastest inference speed of 20.7 FPS, which is 143.5% faster than the 8.5 FPS achieved by the original network.

Comparing the data shown in Table 6 and Table 7, the network lightweight method proposed in this paper has a more obvious speed-up effect on the CPU architecture; at the same time, the practical effect of pruning strategy 2 and pruning strategy 3 is better.

### 4.5. Subjective Effect of Network

**Figure 7:**
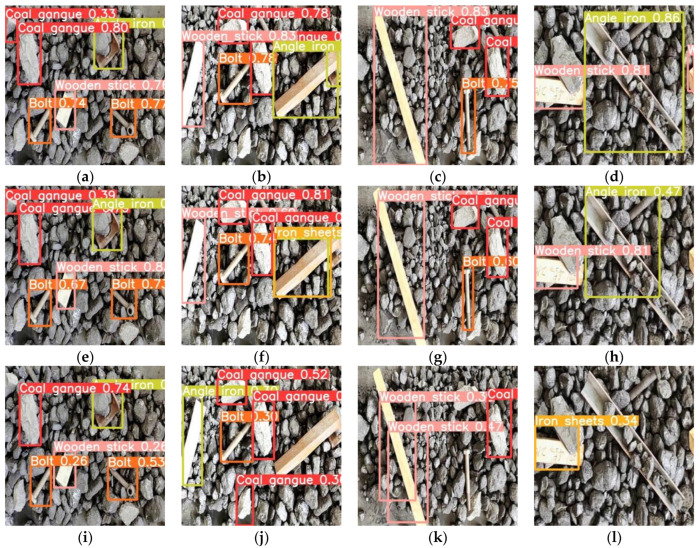
Detection results of the network before and after the improvement in test dataset, (**a**–**d**) belong to the result achieved by the original Yolov5-s network, (**e**–**h**) are the detection results achieved by the improved network when the pruning rate is 0.8, and (**i**–**l**) are the detection results when the pruning rate is 0.9.

The above test results can only describe the performance of networks from a quantitative point of view and cannot visually show the detection effect. Thus, in Figure 7 we have randomly selected four images from the test set of the dataset for the subjective performance description of the networks. In particular, Figure 7a–d shows the detection effect achieved by the original Yolov5-s network, and (e)–(h), (i)–(l) show the detection results achieved by pruning strategy 3 and fine-tuning method 2 when the pruning rate is 0.8 and 0.9, respectively.

In contrast, it can be found that the detection results of the network with a pruning rate of 0.8 are basically similar to the original network, both in terms of the category of the detected target and the positioning of the target; meanwhile, in the network with a pruning rate of 0.9, the detection performance is slightly decreased, which is reflected in the missed detection (Figure 7i,l), false detection (Figure 7j), and inaccurate positioning of the target position (Figure 7k).

**Figure 8:**
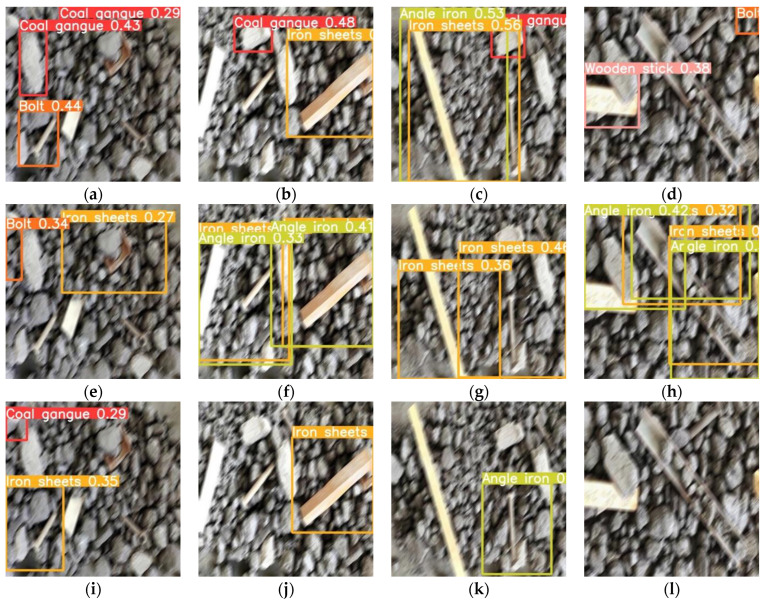
Detection results of blurred images before and after the improvement, (**a**–**d**) belong to the result achieved by the original Yolov5-s network, (**e**–**h**) are the detection results achieved by the improved network when the pruning rate is 0.8, and (**i**–**l**) are the detection results when the pruning rate is 0.9.

Further, we applied a certain amount of motion blur to the images in Figure 7 and again detected the image using the three networks mentioned above, and the results can be found in Figure 8. It can be clearly found that all three networks have a poor resistance to the motion blur, with varying degrees of missed and false detections.

The root cause of the above case may stem from the data level. Because there is no blur information in the input image features of the network, it is impossible for the subsequent feature extraction network to learn such blur features, and naturally it will not make correct judgments or identification on the blurred image information. Therefore, we only need to pass the information of image blur into the network as prior knowledge, and then the effective resistance to motion blur can be realized.

In view of the above, we propose an improvement to the target detection network by means of online data enhancement to pass the prior knowledge. As can be seen from Yolov5, data enhancement techniques such as mosaic and cutmix are applied to images before they are fed into the network for feature extraction, such as stitching and transformations, to enrich the background information and improve the generalization ability of the target detection network. Therefore, in this paper, the image is firstly blurred with the help of the Albumentations tool library with a probability of 0.5 before data enhancement is applied to the image. This online data enhancement approach does not add additional samples to the dataset, nor does it require additional data annotation, and is therefore less laborious and easier to implement.

**Figure 9:**
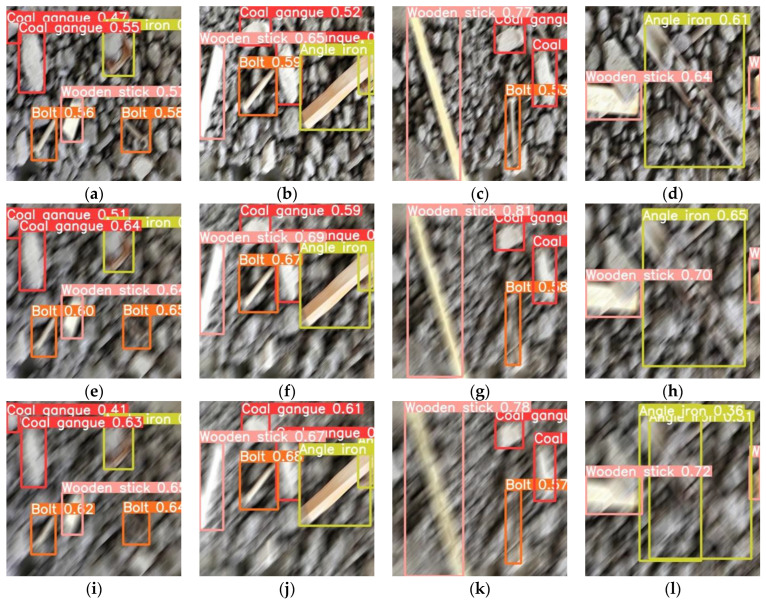
Detection results of blurred images after online data enhancement, (**a**–**d**) belong to the result achieved by the original Yolov5-s network, (**e**–**h**) are the detection results achieved by the improved network when the pruning rate is 0.8, and (**i**–**l**) are the detection results when the pruning rate is 0.9.

Finally, we repeated the above optimization, including retraining and lightening of the network, and re-detected the images in Figure 7 using the same three networks as above, with the results shown in Figure 9. Comparing the images in Figure 7 and Figure 8, the network enhanced with the prior knowledge is more resistant to motion blur and is well suited to images with motion trailing.

### 4.6. Discussion

In this paper, taking foreign object detection as the application scenario, a hybrid compression optimization strategy that integrates network sparse, structure pruning, and knowledge distillation is proposed based on the Yolov5 network, which realizes huge compression for the number of network parameters and calculation amount.

However, considering the inference speed of the optimized network on hardware, the acceleration effect is not very ideal, which may be caused by the following problems, and this is also the focus of follow-up work:

1. Memory factors may lead to different inference speeds. Due to the different frameworks of the three different CPU platforms, the required motherboard types and supported memory dynamic frequencies are different. Therefore, when testing the network inference speed, we actually used three different types of motherboards and memory for the network performance test. The corresponding frequency and size of the memory may have an impact on the network inference speed.

2. The different hardware architectures of CPUs and GPUs may lead to different network acceleration effects after structured pruning, especially the parallel computing framework technology—Compute Unified Device Architecture (CUDA).

3. At the same time, due to the limitation of hardware conditions, we did not run it on real edge computing devices, such as Jetson nano, Jetson NX, and so on. In the future, we will combine the actual situation and further combine weight quantization, Tensor RT acceleration, etc., to accelerate network inference.

## 5. Conclusions

The existence of conveying foreign objects poses a serious threat to the service life of the conveyor belt, which in turn seriously affects the safe and efficient operation of coal mine production. In this paper, considering the needs of the conveying foreign object detection networks running on edge computing devices, based on the Yolov5 network, a hybrid compression optimization strategy that integrates network sparse, structural pruning, and knowledge distillation is proposed. The number of parameters and computation of the delivery foreign object detection network have been extremely compressed, which effectively improves the possibility of the application of the conveying foreign object detection network in edge computing equipment, and also helps reduce the abnormal damage to the belt, thereby reducing unnecessary downtime and production costs and increasing the sustainable capability of coal mines. Compared with previous work, the main contributions and summaries of this paper are as follows:

1. A hybrid compression optimization strategy that integrates network sparse, structure pruning, and knowledge distillation is proposed, which compresses Yolov5’s network parameters and computation by more than 90% and 95%, respectively. The inference speed can be increased by more than 157.5% on three different CPUs and more than 70.3% on three different GPUs.

2. The network fine-tuning method incorporating the knowledge distillation strategy is helpful for the recovery of network performance after pruning and compression, which can stabilize the loss turbulence during the network training process and accelerate the convergence of the network.

3. An online data enhancement strategy has been proposed in this paper with the help of the Albumentations tool library, which improves the network’s resistance to motion blur by means of introducing prior knowledge.

4. Whether the network is lightweight or not cannot be measured simply by the number of parameters and calculations, but should be based on the speed of specific practical reasoning.

## 6. Future Work

In future work, we will address the areas for improvement that were identified in the discussion section to further increase the possibility of applying the proposed algorithm in industrial practice.

## Figures and Tables

**Table 1:** Hardware usage and algorithm operation platform.

Devices				OS	Python	Pytorch
Composition	#1	#2	#3			
CPU	E5-2620V3 (C1)	i7-9750H (C2)	i5-12400F (C3)	Windows10	3.6.13	1.8.0
GPU	RTX 2060 (G2)	GTX 1650 (G1)	RTX 3080 (G3)			
Memory size with frequency	64G-1866 MHz	24G-2666 MHz	32G-3200 MHz			

**Table 2:** Performance of multiple target detection networks in non-coal foreign objects datasets.

Model	Parameter/M	GFLOPs	Model Size/MB	mAP0.5	FPS
CenterNet	32.72	93.20	125	94.58%	23.2
SSD	26.35	52.55	93.2	92.96%	45.3
Yolov3	61.60	123.49	235	91.57%	26.7
Yolov4	64.03	128.43	245	92.14%	22.5
Yolov5-s	7.03	15.9	13.7	98.0%	65.3
Yolov5-m	20.89	48.1	40.8	98.5%	54.5
Yolov5-l	46.16	108	88.5	98.7%	34.9
YoloX-s	8.94	26.65	68.7	88.95%	57.5
YoloX-m	25.28	73.52	193	89.06%	42.7
YoloX-l	54.15	155.33	414	88.07%	37

**Table 3:** The effect of different pruning strategies and different pruning rates on network performance.

Pruning Rate	Pruning Strategy 1		Pruning Strategy 2		Pruning Strategy 3	
	Parameters/M	Model Size/MB	Parameters/M	Model Size/MB	Parameters/M	Model Size/MB
0	7.03	13.7	7.03	13.7	7.03	13.7
0.1	6.16	11.85	5.44	10.5	6.44	12.4
0.2	5.38	10.35	4.37	8.4	5.74	11.05
0.3	4.57	8.8	3.56	6.9	4.92	9.45
0.4	3.66	7.05	2.73	5.3	3.92	7.55
0.5	2.79	5.4	1.95	3.8	2.95	5.7
0.6	2.04	4	1.24	2.45	2.06	4.03
0.7	1.41	2.79	0.71	1.44	1.33	2.63
0.8	0.74	1.5	0.35	0.75	0.61	1.25
0.9	0.13	0.35	0.102	0.28	0.103	0.28

**Table 4:** The effect of different fine-tuning methods on the recovery of prediction accuracy.

Pruning Rate	Pruning Strategy 1		Pruning Strategy 2		Pruning Strategy 3	
	FT 1	FT 2	FT 1	FT 2	FT 1	FT 2
0.1	97.8%	97.7%	96.2%	97.4%	97.7%	97.7%
0.2	97.3%	97.3%	96.3%	97.0%	97.6%	97.6%
0.3	97.1%	97.1%	95.7%	96.5%	97.3%	97.3%
0.4	96.5%	96.7%	95.3%	95.9%	96.7%	96.8%
0.5	96.3%	96.1%	95.1%	95.2%	96.3%	96.3%
0.6	95.4%	95.2%	93.9%	93.8%	95.8%	95.7%
0.7	94.9%	94.3%	91.6%	90.7%	93.9%	94.9%
0.8	92.4%	92.4%	89.3%	88.2%	92.3%	92.1%
0.9	58.4%	80.4%	62.6%	66.2%	78.5%	81.4%

**Table 5:** Computational statistics of the network model after pruning (/GFLOPs).

Pruning Strategy	Pruning Rate								
	0.1	0.2	0.3	0.4	0.5	0.6	0.7	0.8	0.9
1	12.4	10.1	8.4	6.8	5.6	4.7	4.0	3.2	2.4
2	10.0	7.5	5.9	4.8	3.8	3.1	2.5	2.2	1.9
3	13.3	10.9	8.7	7	5.4	4.4	3.6	2.8	2.2

**Table 6:** The inference speed statistics of the pruned network model on different GPUs (/FPS).

Pruning Rate	Pruning Strategy 1			Pruning Strategy 2			Pruning Strategy 3		
	G1	G2	G3	G1	G2	G3	G1	G2	G3
0	52.2	65.3	148.1	52.2	65.3	148.1	52.2	65.3	148.1
0.1	59.3	67.1	150	74.1	70.2	147.1	72.2	69.4	146
0.2	64.7	68.7	151.5	75.8	71.9	145.6	75.3	72.2	148.1
0.3	63.1	69.7	149.3	82.6	68.7	150.4	79.4	67.3	150.4
0.4	63.8	69.2	153.8	82.9	71.2	149.3	86.2	70.9	151.5
0.5	69.0	70.7	152.7	88.1	70.2	152.7	86.6	72.2	152.7
0.6	69.1	71.7	153.8	83.7	71.9	151.5	86.6	69.4	149.3
0.7	69.3	69.0	151.5	85.1	67.6	151.5	86.6	69.7	153.8
0.8	67.3	66.2	153.8	82.6	68.3	151.5	86.2	69.0	153.8
0.9	66.4	66.4	139.9	80.3	67.1	146	88.9	69.0	176.2

**Table 7:** The inference speed statistics of the pruned network model on different CPUs (/FPS).

Pruning Rate	Pruning Strategy 1			Pruning Strategy 2			Pruning Strategy 3		
	C1	C2	C3	C1	C2	C3	C1	C2	C3
0	4.0	3.9	8.5	4.0	3.9	8.5	4.0	3.9	8.5
0.1	4.3	4.3	9.4	5.5	4.7	10.4	4.6	4.2	9.1
0.2	4.8	4.8	10.8	5.5	4.7	10.4	4.6	4.6	10.0
0.3	5.0	5.1	11.5	7.0	5.9	13.7	6.1	5.2	11.6
0.4	5.5	5.4	12.3	7.0	6.3	15.3	6.6	5.5	12.5
0.5	6.3	5.8	13.2	7.8	6.8	15.9	7.0	6.0	13.6
0.6	7.0	5.9	13.9	8.4	7.3	17.1	7.4	6.4	14.8
0.7	7.0	6.3	14.8	8.6	7.6	17.9	7.7	6.8	15.4
0.8	7.3	6.5	15.6	9.2	7.9	18.9	8.4	7.3	17.0
0.9	7.9	7.2	16.3	10.3	8.9	20.7	9.3	8.0	18.8

## Data Availability

The data in this study are available on request from the corresponding author.
